# Cluster and Fold Stability of *E. coli* ISC-Type Ferredoxin

**DOI:** 10.1371/journal.pone.0078948

**Published:** 2013-11-12

**Authors:** Robert Yan, Salvatore Adinolfi, Clara Iannuzzi, Geoff Kelly, Alain Oregioni, Stephen Martin, Annalisa Pastore

**Affiliations:** Division of Molecular Structure, National Institute for Medical Research of the Medical Research Council, London, United Kingdom; University of Bologna & Italian Institute of Technology, Italy

## Abstract

Iron-sulfur clusters are essential protein prosthetic groups that provide their redox potential to several different metabolic pathways. Formation of iron-sulfur clusters is assisted by a specialised machine that comprises, among other proteins, a ferredoxin. As a first step to elucidate the precise role of this protein in cluster assembly, we have studied the factors governing the stability and the dynamic properties of *E. coli* ferredoxin using different spectroscopic techniques. The cluster-loaded protein is monomeric and well structured with a flexible C-terminus but is highly oxygen sensitive so that it readily loses the cluster leading to an irreversible unfolding under aerobic conditions. This process is slowed down by reducing conditions and high ionic strengths. NMR relaxation experiments on the cluster-loaded protein also show that, once the cluster is in place, the protein forms a globular and relatively rigid domain. These data indicate that the presence of the iron-sulfur cluster is the switch between a functional and a non-functional state.

## Introduction

Iron-sulfur (Fe-S) cluster proteins are ubiquitous in nature and perform a wide variety of roles including electron transfer, enzyme regulation and gene expression control [1], [2], [3]. Their biological assembly and maturation is carried out by the Iron Sulfur Cluster (ISC) biogenesis pathway which comprises a complex multi-component machine that, in eukaryotes, is mainly located in mitochondria. Disruption of the ISC pathway occurs in people who suffer from Friedreich’s Ataxia and other mitochondrial diseases [4], [5].

While a main interest stands in understanding the way the ISC machine works in humans, bacteria provide excellent model systems for studying ISC biogenesis. In these organisms the ISC machine is grouped in specialised operons, namely ISC, NIF and SUF each of which operates under the same promoter [3], [6]. Amongst the ISC components are a cysteine desulfurase enzyme, scaffold proteins, the iron binding frataxin, chaperone and co-chaperone proteins, a [2Fe-2S] ferredoxin (Fdx) and other poorly characterised proteins. Direct orthologues in eukaryotes with high sequence conservation include NFS1 (IscS), ISCU (IscU), ISCA1/2 (IscA), FDX1/2 (Fdx), FXN (CyaY), GRP75 (HscA), HSC20 (HscB) and FDXR (FNR) [2].

The presence of Fdx in the ISC machine is particularly intriguing. Ferredoxins possess iron-sulfur clusters which impart redox potential. It has been proposed that they function in electron transfer steps in cluster assembly, though the precise mechanism(s) is/are poorly understood [7], [8]. Ferredoxins involved in cluster formation are of the bacterial [2Fe-2S] class. Prototypic examples include *E. coli* Fdx, *Pseudomonas putida* putidaredoxin and *Rhodobacter capsulatus* FdVI that coordinate the cluster with four cysteines with the consensus sequence Cys-X_5_-Cys-X_2_-Cys-X_n_-Cys [9], [10], [11]. Humans possess two ferredoxins of this class, Fdx1 and Fdx2, which are implicated in steroid biogenesis and cluster assembly [12], [13]. Despite the high sequence similarity between the two, Fdx2 is specific for cluster assembly. Cowan and co-workers have proposed that Fdx2 undergoes a temperature dependent conformational change not observed in Fdx1 that could explain this difference in specificity [14].

In order to elucidate the mechanism(s) of Fdx function, it is necessary to characterise its interactions with ISC components. These include the cysteine desulfurase IscS, the chaperone HscA and the scaffold protein IscA [15], [16]. A prerequisite for these studies is however the possibility of reliably and reproducibly handling the protein. Although the structures of holo-Fdx from different species are well described [9], [17], [18], holo-Fdx presents in fact a number of non-trivial challenges. It is sensitive to oxygen and therefore needs to be handled under anaerobic conditions. Exposure to oxygen causes progressive loss of the Fe-S cluster, giving rise to heterogenous apo/holo-Fdx populations which can prevent or bias a detailed structural/functional characterization of this protein.

Using different techniques that include circular dichroism (CD), absorbance and nuclear magnetic resonance (NMR) spectroscopies, we have thus carried out a detailed investigation of the factors that stabilize the ISC-type Fdx fold from *E. coli* and could allow us to reproducibly prepare homogenous samples that are stable for several days even under aerobic conditions. Our optimised conditions allowed us to obtain the complete backbone assignment of holo-Fdx. We used this information to explore the dynamic properties of the holo-Fdx fold and identify the regions of higher flexibility. Our structural comparison of holo-Fdx with the cluster-free form (apo-Fdx) indicates that, in contrast to holo-Fdx that adopts a stable fold, apo-Fdx is unstructured and gains a fold only upon cluster binding. This finding has implications for structural studies of Fdx and adds another layer of complexity when investigating the interactions of Fdx with the components of the ISC pathway.

## Methods

### Fdx Cloning, Expression and Purification

*E. coli* Fdx was amplified by PCR from *E.* coli genomic DNA with a 5′ NcoI restriction site and a 3′ stop codon and NotI restriction site. This was cloned into a modified pET-24 vector (EMBL Hamburg). The resultant transcript under the control of the T7 promoter codes for the fusion protein 6xHis-GST-Fdx with a Tobacco Etch Virus (TEV) protease cleavage site between the GST-tag and Fdx.

Protein was expressed in BL21 (DE3) cells in LB supplemented with an additional 40 µM Fe(NH_4_)_2_(SO_4_)_2_ to increase the yield of holo-Fdx. Cells were grown at 37°C and induced with 0.5 mM isopropyl β-D-1-thiogalactopyranoside (Generon) at OD_600_ = 0.5–0.7 for 4 hours. For ^15^N labelled and doubly labelled ^15^N/^13^C samples M9 minimal media were used with ^15^N (NH_4_)_2_SO_4_ (15N2, 99%, CIL) and ^13^C D-glucose (U-13C6, 99%, CIL). Clarified cells were lysed by freeze-thaw cycles in buffer containing 20 mM Tris-HCl, 150 mM NaCl, 10 mM Imidazole, 20 mM β-mercaptoethanol, 2% v/v IGEPAL, lysozyme, DNaseI and Complete® EDTA free protease inhibitor (Roche). The resultant cell lysate was clarified by centrifugation at 35,000 *g*.

Protein was purified using Ni-NTA resin (QIAGEN) using a similar protocol described previously [19], [20]. The 6xHis-GST-tag was cleaved using 6xHis-TEV protease (produced in-house) under dialysis in 20 mM Tris-HCl, 150 mM NaCl, 20 mM β-mercaptoethanol, pH 8 overnight at 4°C and then removed by binding to Ni-NTA resin whilst allowing the cleaved Fdx to pass into the wash fraction. Fdx was subsequently concentrated and purified by size exclusion chromatography (SEC) using a 16/60 Superdex G75 column (GE Healthcare) in 20 mM Tris-HCl, 150 mM NaCl, 20 mM β-mercaptoethanol, pH 8. The peak corresponding to holo-Fdx was further enriched by anion exchange chromatography using a 10/10 monoQ column (Pharmacia Biotech). Residual apo-Fdx eluted in a salt gradient at <200 mM NaCl and holo-Fdx at 200–400 mM NaCl. Protein concentration was determined using ε_280_ = 6,990 M^−1^cm^−1^, calculated using ProtParam [21]. The purified holo-Fdx samples had a A_458_/A_280_ value of >0.45 which was taken to be >90% holo-Fdx, based on the reported ε_458_ of iron in the [2Fe-2S] holo-Fdx of 7,071 M^−1^cm^−1^ [22].

### Absorbance Spectroscopy

Absorbance spectroscopy was carried out on a Varian Cary 50 Bio UV-Visible Spectrophotometer. The analysed samples were dissolved in 20 mM Tris-HCl buffer and 150 mM NaCl at pH 8.

### NMR Spectroscopy of Fdx

^15^N SOFAST-HMQC and ^15^N HSQC spectra of ^15^N Fdx were recorded in 20 mM Tris-HCl, 150 mM NaCl, 5 mM TCEP, pH 8 at 298 K. Backbone assignment of ^1^HN, ^15^N, ^13^C^α^, ^13^C^β^, ^13^C’ was facilitated using HNCACB, CACB(CO)NH, HNCO and HN(CA)CO spectra [23], [24] using ^15^N/^13^C labelled holo-Fdx in 20 mM Tris-HCl, 500 mM NaCl, 20 mM TCEP, pH 8 at 298 K under anaerobic conditions in septum sealed 5 mm tubes. Spectra were processed using nmrPipe [25] and analysed with NMRView [26] and an associated assignment module [27]. MARS [28] was used for the first round of automatic assignment, which was validated and completed manually.

^15^N T_1_, T_2_ and ^1^H-^15^N heteronuclear NOE (nuclear Overhauser effect) relaxation data were collected as described [29], [30] at 298 K and 600 MHz in 20 mM Tris-HCl, 500 mM NaCl, 20 mM TCEP, pH 8. T_1_ and T_2_ were calculated using the two parameter fit in the Rate Analysis module and the ^1^H-^15^N heteronuclear NOE was calculated using the hetNOE module in NMRView. The trimmed mean value of R_2_/R_1_ was used to determine the correlation time (τ_m_) using the program r2r1_tm (formerly the tmest routine in the Modelfree program) [30], [31], [32]. Bruker 700 MHz Avance II+, Bruker 600 MHz Avance I or Varian Inova 600 MHz spectrometers with TCI Cryoprobes or HCN probe at the MRC-Biomedical NMR Centre were used for all data collection.

Full assignment was deposited in the BMRB database with the entry number 19273.

### Far-UV CD Measurements

Far-UV CD spectra were recorded on a Jasco J-715 spectropolarimeter fitted with a cell holder thermostated by circulating water from a Neslab RTE-111 water bath. Measurements were carried out on samples in 20 mM Tris-HCl, 500 mM NaCl pH 8 buffer using protein concentrations of 750 µg/ml and QS quartz 0.2 mm demountable cuvettes. CD spectra were typically recorded with 0.2 nm resolution and were base-line corrected by subtraction of the appropriate buffer spectrum. Thermal unfolding curves were obtained by monitoring the ellipticity at 222 nm using 1 mm pathlength cells and a heating rate of 1°C/min for all samples measured in the temperature range 10°C–50°C. The temperature of the sample was measured with a thermocouple immersed in the protein solution. The values of the apparent melting temperature (Tm) were obtained directly from the unfolding curves postulating a two-state mechanism of unfolding and using non-linear regression analysis, as described in detail elsewhere [33]. The spectra were deconvoluted using the SELCON3 algorithm [34]. The experiments were carried out in duplicates using two independent freshly prepared protein batches.

### Disorder Predictions

Predictions were attempted using GLOBPLOT (http://globplot.embl.de/) and DISEMBL (http://dis.embl.de/) and other programs found in the http://www.disprot.org/predictors.php server.

## Results

### Enrichment of Holo-Fdx for Structural Studies

**Figure 1:**
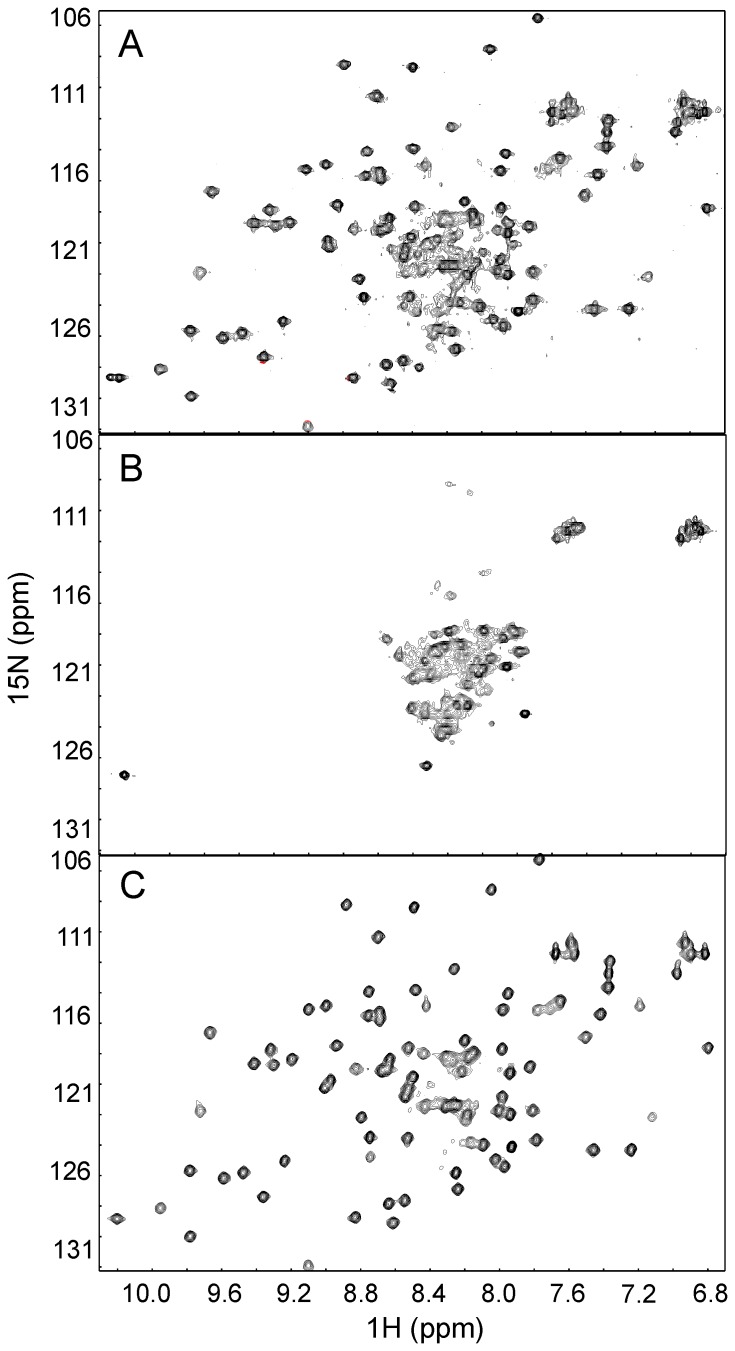
NMR analysis of holo-Fdx purity. A) ^15^N SOFAST-HMQC spectrum of a heterogeneous apo/holo-Fdx after SEC. The presence of more than one population is testified by the number of peaks that is larger than expected and by the overlapping resonances around 8.4 and 121 ppm. The splitted resonance at ca. 10.1 and 129 ppm corresponds to the side chain of the only tryptophan that appears in two conformations. B) ^15^N SOFAST-HMQC spectrum of apo-Fdx acquired after its thermal denaturation. C) ^15^N SOFAST-HMQC spectrum of enriched holo-Fdx following anion exchange chromatography using a monoQ column.

Prerequisite for structural/functional studies *in vitro* is the possibility of obtaining suitable quantities of homogeneous samples. With this aim we first characterized Fdx as produced by bacterial overexpression. Fdx purified by Ni^2+^ affinity chromatography followed by SEC elutes in two partially overlapping peaks which we assumed to correspond to apo- and holo-Fdx (Figure S1). The sample supposedly corresponding to holo-Fdx was pooled and concentrated. It had a faint but clear dark brown colour as expected from the cluster presence [35]. However, NMR analysis revealed that the sample still contains a mixture of species as indicated from the number of resonances in the spectrum in excess of the expected number (Figure 1A). This is likely due to a lack of complete separation of the two species by SEC. Heating the sample to 50°C and cooling down the temperature produced a colourless sample that should correspond to a purely apo-Fdx species: the spectrum at 25°C contains much fewer resonances having lost the set of peaks corresponding to holo-Fdx (Figure 1B). The spectrum is poorly dispersed as it is characteristic of unstructured proteins [36]. An additional purification step of anion exchange chromatography using a monoQ column was included after SEC to enrich holo-Fdx. This step yielded a homogenous sample as determined by the absence of peaks corresponding to apo-Fdx in the ^15^N SOFAST HMQC spectrum (Figure 1C). We followed this protocol for any further characterization of holo-Fdx.

### TCEP and High Salt Attenuate Loss of Fe-S Cluster from Holo-Fdx

**Figure 2:**
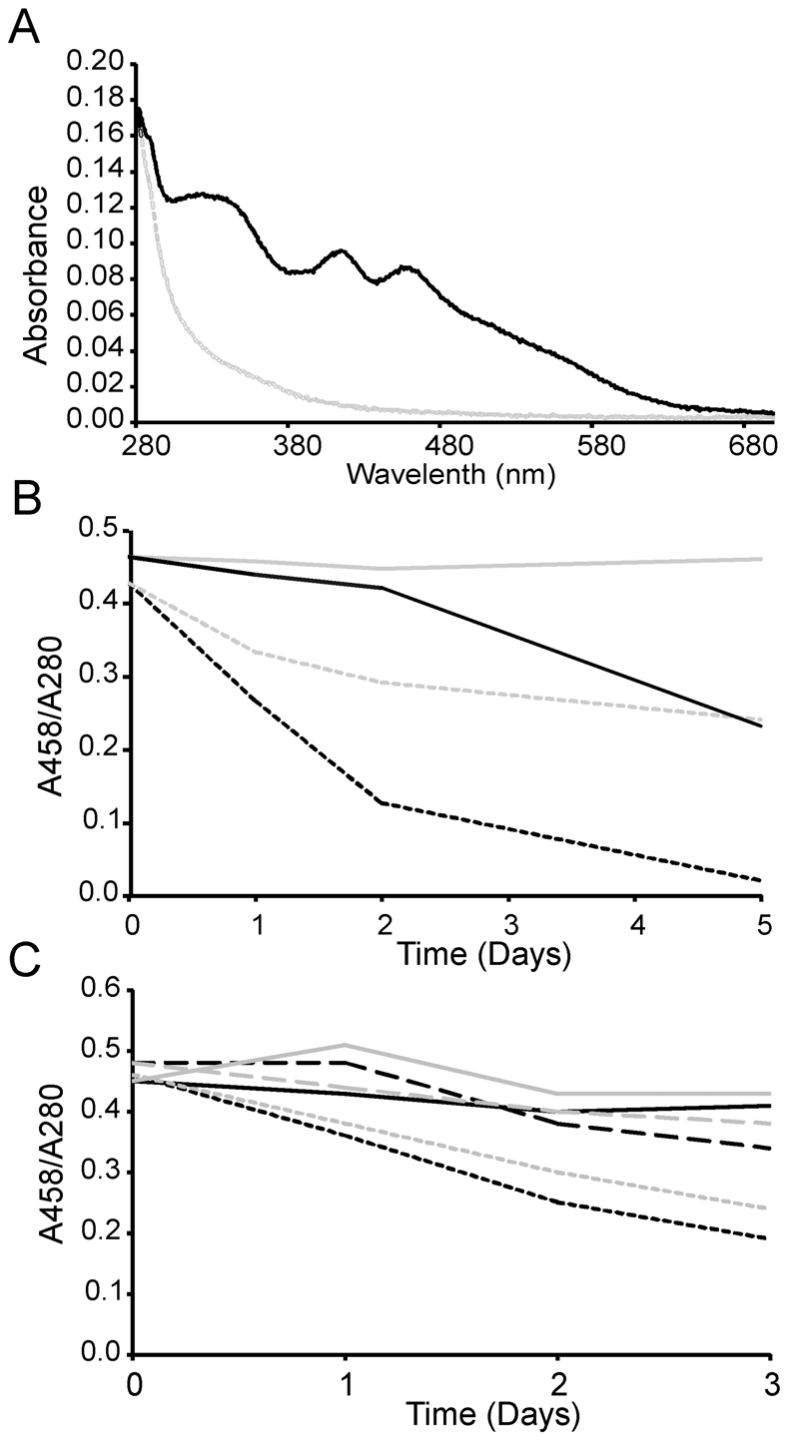
Testing the stability of holo-Fdx. A) Absorption spectra of holo- (black) and apo- (grey) Fdx. B) A_458_/A_280_ measurements for holo-Fdx in 1 M NaCl in the absence (dotted lines) or presence (continuous lines) of TCEP. Anaerobic and aerobic experiments are indicated in grey and black respectively. C) A_458_/A_280_ measurements for holo-Fdx in 20 mM TCEP at 0.05, 0.25 and 0.5 M NaCl concentrations. Anaerobic and aerobic experiments are indicated in grey and black respectively.

We used absorption spectroscopy to monitor the cluster stability on holo-Fdx over time and screen conditions that could increase it. The absorption spectra of apo- and holo-Fdx are distinct (Figure 2A). Calculation of the A_458_/A_280_ ratio gives a rough estimate of the percentage of holo-Fdx in the sample. The presence of TCEP has a dramatic effect on the stability of holo-Fdx and the persistence of the cluster over time. In the absence of TCEP under aerobic conditions, ∼50% of the cluster was degraded after the first 24 hours. Little holo-Fdx remained after 48 hours (Figure 2B). On the contrary, only a moderate loss of Fe-S cluster was observed after 36 hours in the presence of TCEP (Figure 2B). This indicates that TCEP is able to attenuate loss of the Fe-S cluster from Fdx under aerobic conditions likely because it prevents competition between cluster coordination and disulfide formation.

We also assessed the effect of the NaCl concentration on holo-Fdx under aerobic conditions. At concentrations of 500 mM and 250 mM NaCl a moderate loss of Fe-S cluster was observed after 36 hours (<5% and ∼15% respectively) (Figure 2C). At 50 mM NaCl, the loss of cluster was severe (∼45% after 36 hours) even in the presence of TCEP (Figure 2C). This result indicates that high salt stabilises the cluster on holo-Fdx.

### High Temperature Facilitates Loss of Fe-S Cluster from Fdx

**Figure 3:**
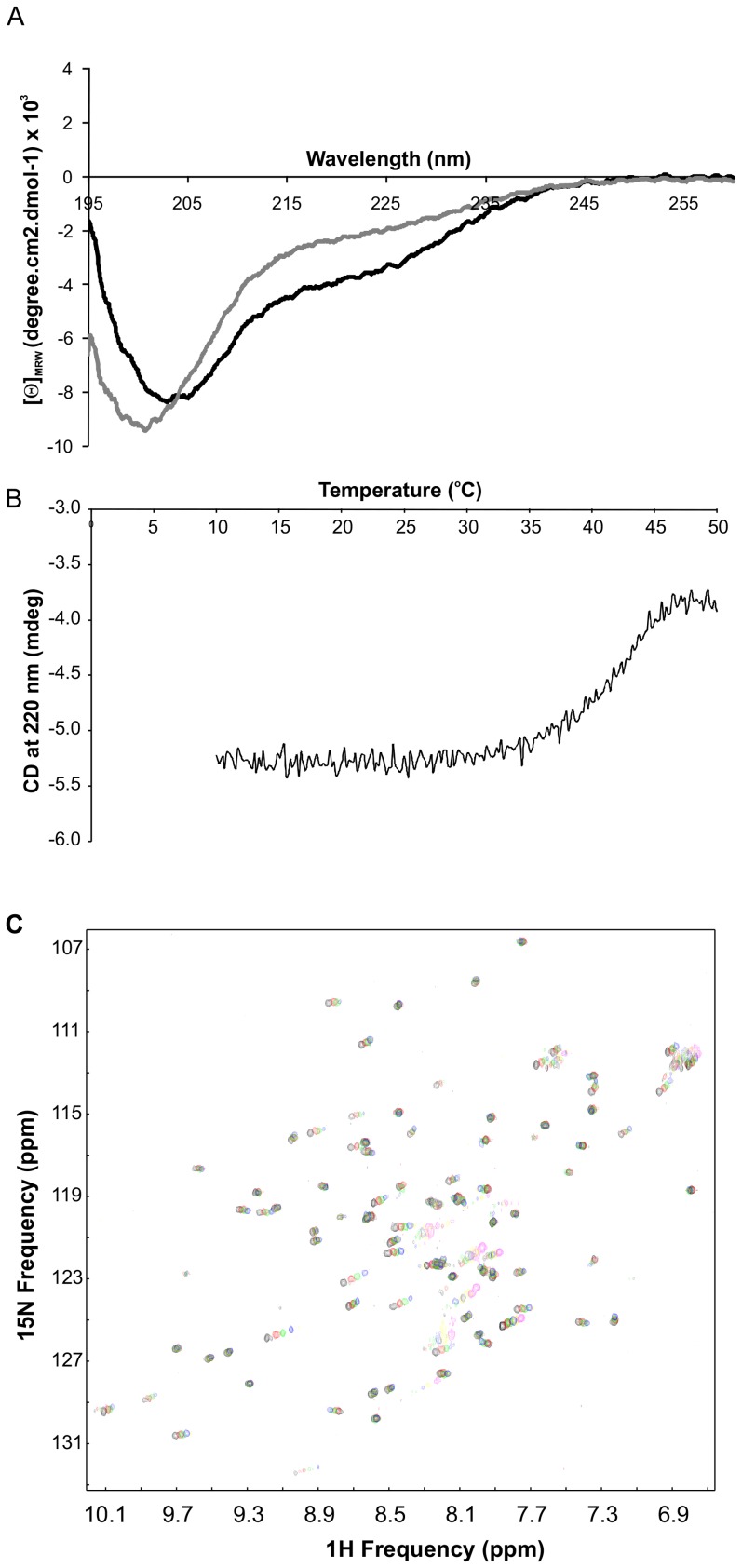
CD analysis of Fdx. A) Comparison of the CD spectra of apo- (grey) and holo-Fdx (black). B) Thermal denaturation curve of holo-Fdx measured at the CD wavelength 220 nm. C) Temperature dependence of the holo-Fdx spectrum. The figure shows the overlay of the ^15^N HSQC recorded at 25°C (black), 30°C (red), 35°C (green), 40°C (blue), 45°C (yellow) and 50°C (magenta).

The CD spectrum of apo-Fdx is typical of an unstructured protein in contrast to that of holo-Fdx in agreement with the NMR data (Figure 3A). The spectrum of freshly prepared holo-Fdx contains two bands around 203 and 220 nm as expected from the αβ mixed Fdx fold [9]. The band at 203 nm is however much more intense than that at 220 nm suggesting the presence of an appreciable random coil contribution. Estimates of the secondary structure content suggest 9% α helix, 16% turn, 24% β sheet and 51% random coil that should be compared with 15% α helix, 22%, 23% β sheet and 40% random coil expected from the X-ray structure [9]. We also investigated the thermal denaturation of holo-Fdx using CD at 220 nm (Figure 3B). Denaturation of holo-Fdx occurs at about 45°C and is an irreversible process. The absorption spectrum after thermal denaturation was characteristic of apo-Fdx indicating that thermal denaturation of holo-Fdx is associated with loss of the Fe-S cluster thus explaining why thermal denaturation is irreversible.

We investigated further using NMR whether holo-Fdx undergoes a conformational change with increasing temperature, as was observed for human Fdx2 [14]. We recorded ^15^N HSQC spectra for holo-Fdx at physiological salt concentration at 25°C, 30°C, 35°C, 40°C, 45°C and 50°C following incubation for 1 hour at each temperature. We observed directionally uniform shifts of the ^15^N HSQC peaks up to 40°C (**Figure 3C**). At 45°C the spectrum resembled a mixture of holo- and apo-Fdx, indicating partial degradation of the cluster. At 50°C the spectrum retained peaks of apo-Fdx only, indicating complete loss of the cluster. This behaviour is at variance to that observed for human Fdx2, where increasing the temperature caused non-uniform peak shifts in different directions [14]. This indicates that *E.coli* Fdx does not undergo a temperature dependent conformational change.

### Chemical Shift Assignment of Holo-Fdx

The possibility of reproducibly preparing homogeneous and stable samples of holo-Fdx allowed for the unambiguous chemical shift assignment of the holo-Fdx NMR spectrum. We decided to record the spectra in 500 mM NaCl although high salt concentrations reduce the signal-to-noise ratio in NMR measurements using cryoprobes [37]. However, we reasoned that a 15% loss of cluster at 250 mM NaCl would not justify the relatively limited gain in signal to noise achieved at 250 mM compared to 500 mM.

**Figure 4:**
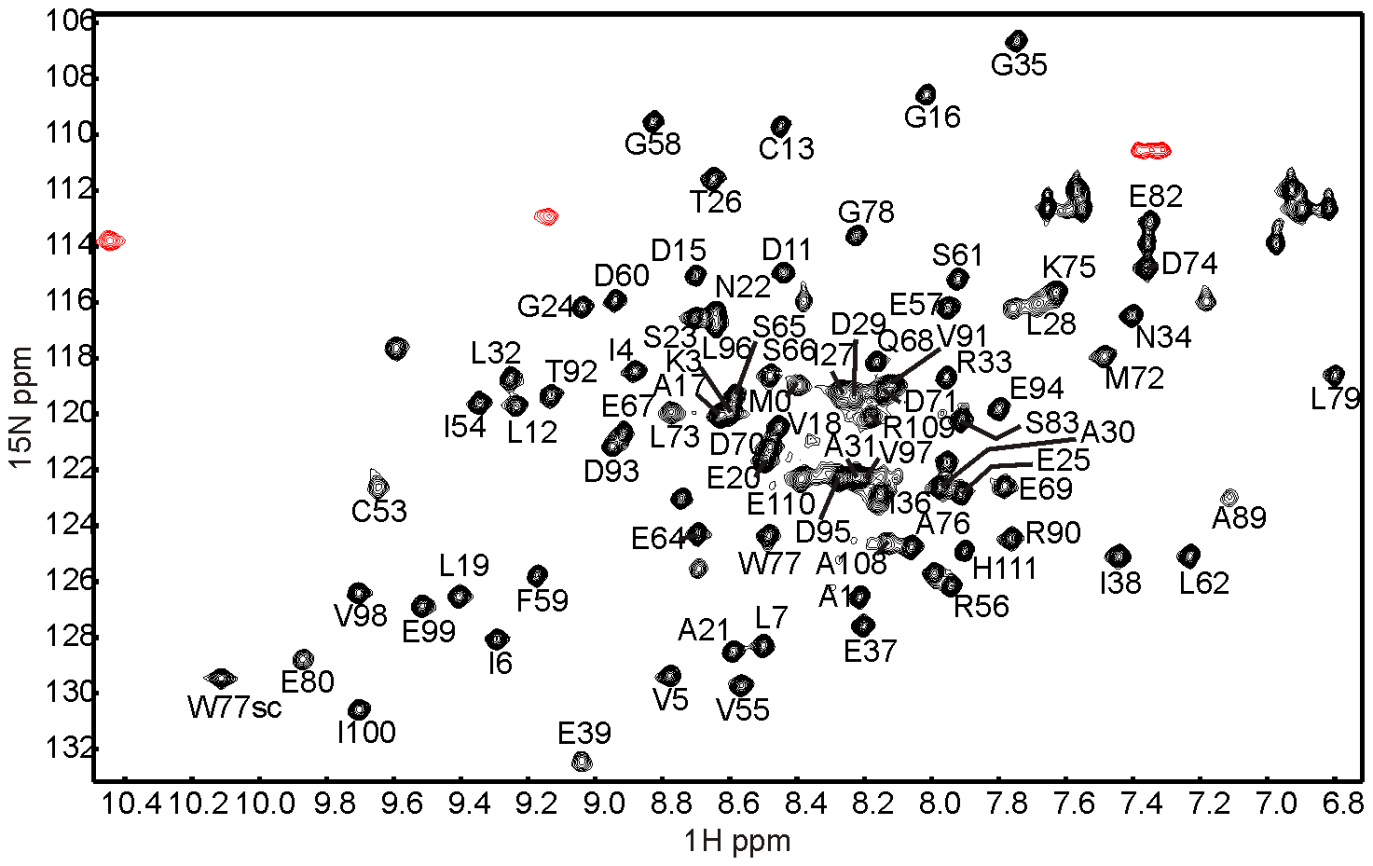
^15^N HSQC of holo-Fdx showing the assigned residues. SC denotes the tryptophan indole side chain N^ε1^H^ε1^ group. Folded peaks are shown in red.

Spectral assignment of 72% of the backbone HN, C^α^, C^β^ and C’ atoms was achieved by a combination of automated and manual assignments (Figure 4). Residues 40–52, 84–88 and 102–107 could not be assigned. The first two stretches of residues are positioned around the [2Fe-2S] cluster co-ordination site. Therefore, their chemical shifts were most likely unobserved because of the paramagnetic relaxation experienced in close proximity to the cluster. This behaviour was also observed in the NMR assignments of bovine adrenodoxin [38] and in other cluster-loaded proteins. Residues 102–107 are located at the protein C-terminus. This region has been shown to undergo conformational changes upon oxidation/reduction of the [2Fe-2S] cluster in bovine adrenodoxin, thus indicating flexibility [38].

### The Dynamic Properties of Holo-Fdx

**Figure 5:**
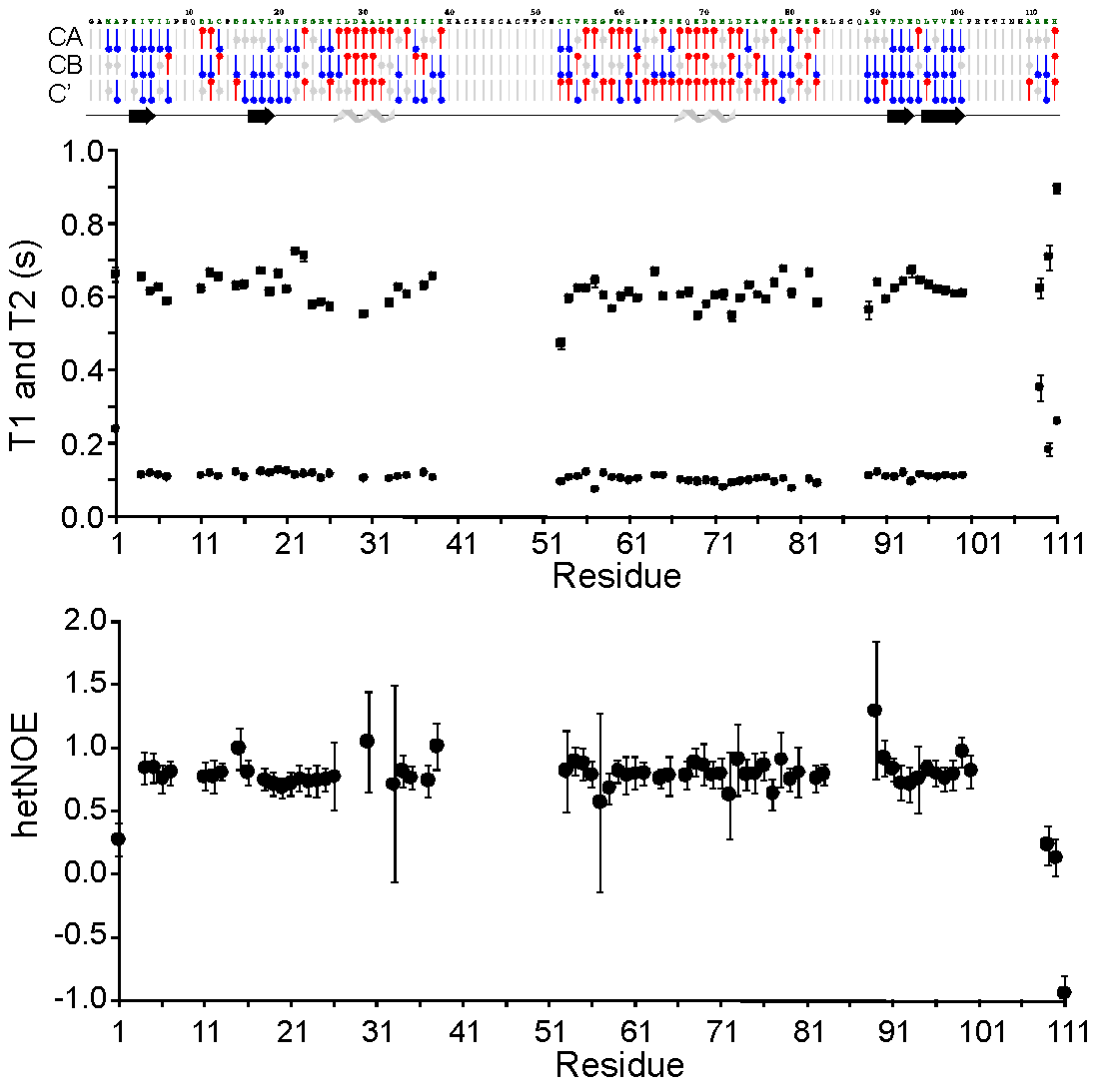
Structural and dynamic characterization of Fdx. Chemical Shift Index analysis of CA, CB and C’ of holo-Fdx. Assigned residues are coloured green. Red “lollipops” denote helical chemical shifts, blue “lollipops” denote β sheet chemical shifts. Secondary structure is shown under the CSI plot where there is a consensus. B) NMR relaxation parameters.

We used the spectral assignment to ensure the structural integrity of the protein and to further characterize the backbone dynamics of holo-Fdx by recording NMR relaxation experiments at 298 K and 600 MHz (Figure 5). A plot of the chemical shift indexes [39] shows the distribution of secondary structure elements along the sequence. This is in good agreement with what observed in the crystal structure [9] showing also long unstructured regions. The average T_1_ and T_2_ are around 620 ms and 110 ms respectively with minor deviations in loop regions. The heteronuclear NOE values are also fairly flat with an average around 0.8. Significant deviations from these values occur at the C-terminus only, indicating a well structured and ordered domain with only marginal flexibility at the C-terminus. A similar behaviour was previously reported for bovine adrenodoxin [38]. A correlation time of 6.9 ns ±0.09 was calculated from the trimmed mean R_2_/R_1_ value. This value is compatible with holo-Fdx being monomeric in accordance with the molecular weight dependence of the overall rotation of a molecule, expected from the Stokes-Einstein-Debye equation [40].

## Discussion

We have characterized the factors that determine the fold stability and the dynamic properties of Fdx, an *E. coli* adronedoxin-type ferredoxin involved in iron-sulfur cluster biogenesis. Understanding the determinants of protein fold is the prerequisite for structural and biophysical studies and for correctly correlating them to function. Stability studies are particularly important with metallo-proteins since recombinant expression and purification of these proteins at the concentrations and homogeneity needed for biophysical characterization is often challenging due to the complex specific post-translational maturation processes that influence their fold. Full characterization is thus always essential to avoid preparations that may contain significant fractions of inactive apo-proteins.

In the case of Fdx, stability has a two-fold meaning: it refers to the fold stability as well as to the tendency to lose the cluster. The two concepts are strongly intertwined.

**Figure 6:**
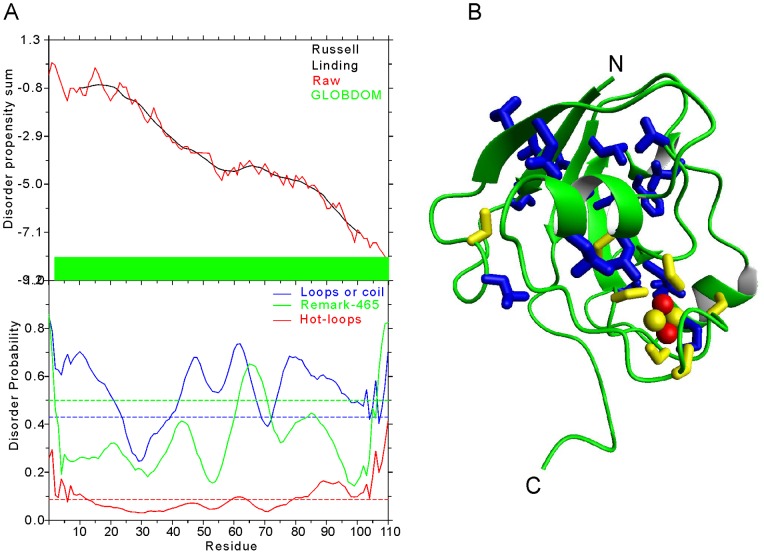
Disorder predictions and holo-Fdx fold. A Predictions obtained by GLOBPLOT (top) and DISEMBL (bottom). B) Ribbon representation of the crystal structure of holo-Fdx (PDB code 1I7h) using Pymol Version 1.4.1. The fold is compact and wraps around the cluster (indicated in yellow and red). The side chains of cysteines are indicated in yellow, hydrophobic side chains (Met, Leu, Ile, Val and Phe) are indicated in blue.

We have shown that, while still present in the overexpressed protein, the iron-sulfur cluster is readily lost under aerobic conditions reflecting high oxygen sensitivity. The apo protein is devoid of tertiary structure when in the absence of the prosthetic group as supported both by the CD and NMR spectra. This however does not make apo-Fdx an IUP [41]. We wondered whether the observed properties of apo-Fdx would classify Fdx as an intrinsically unstructured protein (IUP). It is only in the last ca. 10–15 years that the scientific community has widely recognized that not all proteins are globular [41]. Some are devoid of intrinsic structure but fold only upon specific conditions or remain unfolded to fulfil a role as linkers and/or as elastic connectors. Several servers have been developed that predict and recognize IUPs. We used two of the most widely used servers to understand whether what we observe by NMR on the apo protein reflects an intrinsic sequence signal (Figure 6A). GLOBPLOT suggests the presence of a unique globular domain since downhill plots correspond to putative globular regions, whereas uphill regions predict protein disorder. Likewise DISEMBL, which describes protein disorder as two-state models where each residue is either ordered or disordered, suggests the presence of extensive loops (blue plot above the dashed blue line threshold) but gives low probability for loops with a high degree of mobility and of missing coordinates in X-Ray structure as defined by REMARK-465 entries in PDB. Other prediction programs are fully consistent with these results indicating that the Fdx sequence is in no way recognized as an unstructured protein.

Accordingly, when cluster loaded, Fdx behaves as a compact and relatively rigid globular protein as proven by the dynamics of the holo-protein observed by our NMR relaxation data. A relatively high flexibility is observed only near the protein C-terminus. While being fully consistent with the Fdx crystal structure which shows a compact fold that wraps around the cluster (Figure 6B), these data indicate an essential role for the cluster in determining and stabilising the fold. A similar behaviour has been documented also for other Fdxs from mesophile organisms [42], [43], [44]. In agreement with the importance of the cluster for the Fdx fold is the evidence that replacement of any of the cysteine ligand residues in adrenodoxins with serines leads to the formation of apo-proteins [45], [46]. What we observe is strongly reminiscent of other proteins that may fold only under the influence of post-translational modifications. Well known examples are the zinc finger family in which fold is determined by zinc binding or the globin family some of whose members form molten globule states in the absence of the stabilising haem while become stably folded compact proteins in the presence of the prosthetic group [47], [48]. An obvious consequence of these observations for Fdx is that, *in vivo*, cluster insertion must precede folding of the Fdx polypeptide. It also brings attention to the limitations of using servers to predict protein fold states. It is for this reason that we made a point in demonstrating how sequence predictions cannot predict the behaviour of Fdx: although with a sequence distribution compatible with a folded protein Fdx is unfolded in the absence of a prosthetic group. Specific identification of this subclass may be interesting both from the point of view of protein classification [49] and for the functional importance of this distinct family of proteins.

We searched for conditions that might stabilise the Fdx fold under aerobic conditions and slow down cluster detachment. We observed that strong reducing conditions as well as high ionic strengths are key elements for enhancing cluster stability. The first effect may be explained by a competition between cluster coordination and disulfide formation. The second is probably due to an entropic effect of salts on the overall fold stability. The observed behaviour is in excellent agreement with what observed with spinach Fdx [44]. This high oxygen sensitivity is shared, in the ISC operon, also by the scaffold protein IscU and is probably related to the necessity of this machine to be highly sensitive to oxidative stress [1], [2], [3].

Our studies highlight an aspect that could have important implications for the structural and functional characterisation of Fdx in the context of the ISC pathway: it might be of interest to wonder how the unstructured/structured states of apo- and holo-Fdx respectively may relate to the functions of the protein *in vivo*. An easy hypothesis is that the different conformational properties of apo- and holo-Fdx could have evolved to allow a differentiation between the interactions of apo- and holo-Fdx with other cellular components. Our preliminary data show for instance that holo-Fdx but not apo-Fdx binds to the IscS desulphurase [50]. These findings, together with the recent demonstration that ISC Fdx plays a reducing role in the early events of cluster formation [7], [8], provide us with the first direct information on the mechanism of functioning of this protein.

Our work thus provides the foundations for further structural and/or quantitative characterization of Fdx and for probing interactions with other components of the ISC pathway. Under this perspective, we have indicated once again the importance of NMR in assessing protein folding and determining sample homogeneity. Further work will be needed to understand the precise role of the Fdx protein in ISC biogenesis and the significance of the different interactions which it may be involved in.

## Supporting Information

Figure S1 **Elution profile of holo-Fdx on a 16/60 Superdex G75 column.** A280 and A458 profiles are shown in blue and red respectively. Holo-Fdx eluted at 87 ml as indicated by the increase in A458 which represents the [2Fe-2S] cluster. Apo-Fdx elutes at 76 ml as determined by SDS-PAGE and NMR analysis.
(PDF)
