# Supplementary material for: Cluster and Fold Stability of E. coli ISC-Type Ferredoxin
Source: PLoS One. 2013 Nov 12;8(11):e78948. doi: 10.1371/journal.pone.0078948 (PMC3827102; doi:10.1371/journal.pone.0078948)
Supplement: Figure S1 — Elution profile of holo-Fdx on a 16/60 Superdex G75 column. A280 and A458 profiles are shown in blue and red respectively. Holo-Fdx eluted at 87 ml as indicated by the increase in A458 which represents the [2Fe-2S] cluster. Apo-Fdx elutes at 76 ml as determined by SDS-PAGE and NMR analysis. (PDF) [file pone.0078948.s001.pdf]

Supplementary Materials

Figure S1. Elution profile of holo-Fdx on a 16/60 Superdex G75 column. A_{280} and A_{458} profiles are shown in blue and red respectively. Holo-Fdx eluted at 87 ml as indicated by the increase in A_{458} which represents the [2Fe-2S] cluster. Apo-Fdx elutes at 76 ml as determined by SDS-PAGE and NMR analysis.

Figure S1

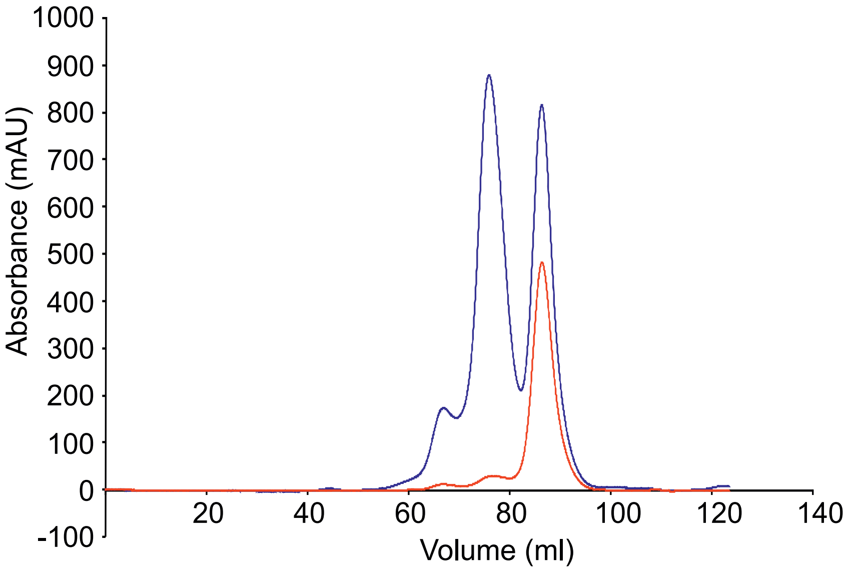

1000
900
800
700
600
500
400
300
200
100
0
-100
Absorbance (mAU)
20
40
60
80
100
120
140
Volume (ml)
